# Addenda to Mr. Freake's Paper on the Humulus Lupulus

**Published:** 1807-11

**Authors:** 


*Addenda to Mr. Freake's Paper on the Humulus Lupulus.*

WHEN I had for more than four years prescribed the Humulus Lupulus of Linnæus, with apparent advantage in several cases of Gout, and other disorders, particularly during the continuance, and after the influenza of 1803, and found that it possessed very valuable properties, I published my first account of its virtues in the Medical and Physical Journal for May, 1805, with the hope that practitioners might be induced to try the medicine, and that under their direction it would be found to produce similar effects.

From this time I continued to make observations respecting the powers of this vegetable, when any fair opportunity offered, and seldom a week passed without affording some additional proof to confirm my opinion of the utility of the medicine. I became anxious that my observations should be beneficially extended, and for that purpose I collected the opinions of several authors, which, with observations, chemical experiments, (both from the green and the dried flowers) and the addition of eight cases, I formed into a pamphlet, which was printed in May, 1806, and entered at Stationers' Hall; yet I did not allow any of this edition to be sold, for it was my wish that the preparations should be sanctioned by the College of Physicians before they were given to the public. This pamphlet was therefore intended solely for the use of professional gentlemen.

Several physicians prescribed the Lupulus with beneficial effect, though only four favoured me with their opinions for publication, which with the two additional cases, No. 9, and 10, (certainly of considerable importance*), and some remarks, and directions, comprized the second edition, in which, for the reason before assigned, the chemical experiments were omitted.

I think it proper to notice, that whenever I had opportunity of enquiring, I found that the people employed during the process of collecting and preparing the Hops for sale, had good appetites, slept well, and were remarkably healthy; this circumstance will probably be admitted as an

* The latter is now rendered more so, on account of the patient having had another violent attack in July last, when the Lupulus was again taken, and he was once more restored by it to a good state of health.

an additional proof to strengthen my opinion of the value of the medicine.

I shall now offer my best acknowledgments to the Editors of the different Reviews, who have thought proper to notice these pamphlets, for the handsome manner in which they have generally spoken of them, and request they will accept such acknowledgment as a token of my ardent desire to promote public good.

I shall thankfully receive any friendly hint that has a tendency to confirm the good effects of this medicine, and as it is supposed by those who have not seen the first edition, that the second edition is defective, in omitting to say how the tincture and extract were to be obtained, I take this opportunity to inform medical gentlemen, that the tincture I prepare, is in the proportion of a pound and a half of the dried flowers of the Lupulus to a gallon of the purest proof spirit, occasionally using steam heat, and I reduce the alkohol to proof strength with an infusion made from the ingredients of the preceding tincture. The extract is the aqueous preparation of the dried flowers of the Lupulus, made in the usual manner directed for extracts by the London College.

I have preferred using these preparations, because one gallon of this tincture affords nine drachms more extract after the spirit is drawn off, than the tincture prepared with alcohol, and a considerable less quantity of spirit is taken by the patient. And the aqueous extract seems better adapted for medical use than the spirituous, inasmuch as the proportion obtained is as eight ounces of the aqueous, which is easily dissolved in any weak liquid, to five ounces of the resinous extract, which after it is with some difficulty dissolved, soon after separates and adheres to the sides of the vessel. This resin is acted on by atmospheric air; so much so, that it cannot be reduced to powder like others.

I am truly sorry to remark that complaints have already been made of the tincture and extract, as prepared at some houses in London; and as so much depends on the preparation of the medicine, I have taken care that the faculty, and the public at large, may be supplied with both the preparations at my residence, as it may probably be a considerable time before the new Pharmacopœia is published, when, if these preparations should be adopted, every medical house will be expected to keep both tincture and extract ready for use, and subject to be inspected by the Censors of the College.

